# Exposure Science in the 21st Century: Advancing the Science and Technology of Environmental Sensors through Cooperation and Collaboration across U.S. Federal Agencies

**DOI:** 10.3390/chemosensors8030069

**Published:** 2020-08-13

**Authors:** Janis Hulla, Vasu Kilaru, Gregory Doucette, David Balshaw, Tim Watkins

**Affiliations:** 1 Sacramento District, United States Army Corps of Engineers, EDE, 1325 J Street, Sacramento, CA 95814, USA; 2 Center for Environmental Measurement & Modeling, Office of Research and Development, United States Environmental Protection Agency, Mail Drop D343-02, 109 TW Alexander Drive, Durham, NC 27709, USA; 3 NOAA/National Ocean Service, National Centers for Coastal Ocean Science, 219 Fort Johnson Rd., Charleston, SC 29412, USA; 4 Exposure, Response, and Technology Branch, National Institute of Environmental Health Sciences, 530 Davis Dr. suite 3006, Morrisville, NC 27560, USA; 5 Center for Environmental Measurement and Modeling, Office of Research and Development, United States Environmental Protection Agency, Mail Drop D305-01, 109 TW Alexander Drive, Durham, NC 27709, USA

**Keywords:** ES21, environmental monitoring, real-time, chemical sensors, mobile sensors

## Abstract

The convergence of technological innovations in areas such as microelectronics, fabrication, the Internet-of-things (IoT), and smartphones, along with their associated “apps”, permeates many aspects of life. To that list we now can add environmental monitoring. Once the sole purview of governments and academics in research, this sector is currently experiencing a transformation that is democratizing monitoring with inexpensive, portable commodities available through online retailers. However, as with any emerging area, several challenges and infrastructural hurdles must be addressed before this technology can be fully adopted and its potential be realized. A unique aspect of environmental sensing that differentiates it from some other technology sectors is its strong intersection and overlap with governance, public policy, public health, and national security—all of which contain some element of inherent governmental function. This paper advocates for and addresses the role of sensors in exposure science and illustrates areas in which improved coordination and leveraging of investments by government have helped and would catalyze further development of this technology sector.

## 1. Introduction

To meet the challenges associated with implementing environmental sensor-based applications, many U.S. federal agencies have initiated efforts to understand the capabilities of these technologies to support their mission and are aiding the overall development of this technology sector through research, grants, and contracts. At the request of the Environmental Protection Agency (EPA) and the National Institute of Environmental Health Sciences (NIEHS), the National Academy of Sciences Medicine and Engineering (NASME) published a report in 2012, titled *Exposure Science in the 21st Century: A Vision and Strategy* [1]. With this report as impetus, The Federal Working Group on Exposure Science in the 21st Century (ES21 FWG) [2] was conceived and convened. The charter of the “Sensors and Dosimeters” subgroup of the ES21 FWG was to review the recommendations of the report, inventory current and emerging tools, identify major gaps, and develop options for new approaches that may enhance or replace older approaches. Despite the broad range of applications for these technologies, there is general agreement within the federal government’s scientific enterprise that the public sector plays a role in supporting the use and evaluation of sensors, and in educating developers and potential users about their responsible and effective uses.

Given the breadth of scientific disciplines that comprise exposure science, the NASME report asserted that greater coordination across scientific disciplines, as well as across the federal government, was necessary in order to incorporate advancements in science and technology already being used in related domains, such as genomics and toxicology, into exposure science. Advancements in computational methods [3], high-throughput screening techniques [4,5], the aggregation, integration, and analysis of multiple data streams (big data), and use of new and novel technologies (including sensors) have impacted the evolution and capabilities of exposure science in recent years and offer even greater promise in the future. In addition, implementing infrastructural elements such as data standards, ontologies, and the provision of linked open data with computable semantics are necessary to realize the NASME’s broader vision for advancing exposure science.

Any practical implementation of the concept of the “exposome” [6,7] (the totality of exposures from prenatal to death) necessitates broad changes in the manner in which scientific research is conducted and shared [8]. While the idea of quantifying the exposome for every possible exposure may be impractical to nearly impossible in many cases with today’s state of the science, the use of sensor technologies for personal exposure monitoring may allow us, in the near future, to come close to quantifying an exposome for a limited number of chemical stressors.

## 2. Current Approach

While a host of factors affect the development and advancement of technologies, not the least of which are market-driven forces, the public sector can have a real and substantial impact in fostering technology development. Awarding federal research grants is one of the primary ways the government prioritizes and influences research and investment in new technologies and ensures information about advancements is publicly available.

All U.S. federal agencies with budgets greater than $100 million are required to participate in the Small Business Innovative Research (SBIR) program and those with budget in excess of $1 billion must also support Small Business Technology Transfer (STTR) grants to foster the development of commercial products. Each agency has its own focus and priorities, with relatively little coordination across agencies. However, several agencies have encouraged efforts focused on the development of technologies for exposure assessment. Because of the size of these agencies and the diversity of interests within and among them, the Sensors and Dosimeters subgroup of the ES21 FWG initiated a single point of entry to access sensor-related SBIR/STTR announcements across federal agencies as a way to make these investments more coordinated and efficient for both potential grantees and grantors [9]. The overall goals of this effort were to: (1) articulate a common need for and interest in sensor technology development, evaluation, and application across the federal government; (2) clarify the federal commitment to sensor technology research and quantify federal SBIR investment; (3) identify potential collaborative investments and avoid duplicative investments; and, (4) create one central web location to identify solicitations and simplify the application for federal funds related to sensor technology for small business innovators.

Given that the mission and scope of federal agencies sometimes overlap, it is instructive to examine below several cases where the coordination and leveraging of common interests and federal partnerships has yielded valuable outputs and outcomes. The My Air My Health (MAMH) challenge provides an excellent example of such collaboration [10] with the aim of conducting a jointly sponsored prize competition involving three agencies: the EPA, the Department of Health and Human Services—Office of the National Coordinator on Health Information Technology, and the NIEHS. The long-term goal was to fulfill a shared mission in public health research, technology development, and demonstration at the intersection of mHealth (mobile health) and environmental monitoring. The MAMH challenge funded an effort to bring together the two data streams that offer great promise in understanding and quantifying the connection between exposure and potential health outcomes. The ability to simultaneously monitor physiological and health parameters (e.g., heart rate, blood oxygen levels, etc.), along with data on environmental conditions (e.g., air pollution or water quality), has the potential to revolutionize environmental epidemiology and precision medicine. Efforts like this illustrate the advantages of coordinated government investments [11].

In collaboration with the Centers for Disease Control and Prevention (CDC) and the National Institute for Occupational Safety and Health (NIOSH), the U.S. Army’s SBIR program is seeking to integrate biomarker data with sensor-derived data to develop a naphthalene dosimeter. The aim is to collect real-time concurrent personal exposure and biomarker data using radiation dosimetry as a paradigm to model internal dose. While the Geiger-Muller tube was the first to measure real-time exposure of a physical entity (i.e., ionizing radiation), the naphthalene dosimeter is the first attempt to transition a real-time chemical sensor into a *dosimeter* [12-14]. This work, which progresses from simply measuring the external concentration to the actual estimation of internal “dose”, represents a significant achievement in technology that can now inform the science for exposure to naphthalene.

Fires, both wildland and prescribed burns, cross agency jurisdiction and are challenging on many fronts. They often occur in areas that are somewhat remote, making monitoring the smoke plumes from the fires a difficult task that has confounded agencies involved in managing and fighting wildfires. The degradation of air quality from wildland fire smoke affects not only the public at large but also the firefighters and others in the field incident teams. Since traditional monitors are too large and expensive to deploy in such a dynamic situation, lower-cost sensors offer some promise for addressing these issues. The 2017 Wildland Fire Sensors Challenge [15] was a multi-agency effort that included the EPA, National Aeronautics and Space Administration (NASA), National Oceanic and Atmospheric Administration (NOAA), National Park Service, the U.S. Forest Service, CDC and a non-profit called Tall Timbers. The main goal of this challenge grant was to develop a low-cost, multi-node air sensor system that is useful for air resource advisors (ARAs) during wildland fire events.

Among the agencies active in the “sensor” arena are NIOSH and the NIEHS. NIOSH’s Center for Direct Reading and Sensor Technologies [16] works with industries, labor, trade associations, and academia to foster advancements in the development and use of sensors for occupational safety and health. Through this virtual center, NIOSH coordinates a national research agenda that supports evaluating the performance of new sensor technologies, training activities, and developing guidance on the selection, use and interpretation of data from direct reading devices. Over the last fifteen years, the NIEHS’s Exposure Biology program has also invested in dozens of projects to develop and validate sensor technologies through grants to researchers working on novel sensor devices. The objective of the NIEHS focus on sensors is to support technology developments that, in turn, can inform exposure science and environmental health in epidemiology, precision medicine, and citizen science [17,18].

The EPA’s Office of Research and Development, through the Air and Energy (A & E) research program, has also made substantial investments in environmental sensing [19]. The EPA has engaged in a wide variety of activities, including: (1) providing resources through its Science to Achieve Results (STAR) grants for research and development and community applications of newer technologies, (2) evaluating the performance of commonly available low- and mid-cost sensors, (3) developing in-house sensor systems (Village Green, Citizen Science Air Monitor CSAM), (4) conducting numerous training sessions and education outreach efforts for users, (5) coordinating with other entities, both nationally with California’s Air Quality Sensor Performance Evaluation Center (AQ SPEC) [20] and internationally with the European Union’s Joint Research Center (EU JRC) [21,22], and (6) participating with non-governmental organizations’ efforts in establishing infrastructural capacities for the air sensor arena [23].

A prime example of a multilateral public-private partnership involving several federal agencies was the nutrient sensor challenge, which brought together numerous governmental and non-governmental entities interested in a common goal. The challengers included the White House Office of Science and Technology Policy, EPA, U.S. Geological Survey (USGS), NOAA, National Institute of Standards and Technology, Department of Agriculture, Alliance for Coastal Technologies, Everglades Foundation, Tulane University, and American University [24]. The idea driving the formation of this partnership is to use the challenge process to spur innovation in areas with critical needs while also involving various interested parties to help design and contribute to the challenge. This challenge attempts to find innovative and low-cost solutions to the problem of identifying and quantifying nutrient loadings in waterways (e.g., lakes, rivers) in order to aid in managing nutrient pollution and its impacts such as algal blooms. NOAA’s National Phytoplankton Monitoring Network (PMN) is a partnership specifically established to monitor algal blooms. This is a community-based network of volunteers monitoring marine and freshwater phytoplankton, harmful algal blooms (HABs) and other water quality parameters. PMN activities facilitate the interrelationships between humans and coastal ecosystems while providing volunteer citizen scientists with meaningful opportunities for hands-on science engagement. These citizen scientists collect important data for species composition and distribution. The Network provides a critical cost-effective infrastructure for deploying new field-portable sensor technologies as they become available. The data on HABs and other important water quality information generated by PMN volunteers represent actionable results that can support decision making by resource managers and public health officials.

The same public–private “challenge” approach is being adopted to seek new design solutions for rapid, cost-effective sensors capable of detecting the activation of one or more toxicity pathways while deployed in aquatic systems. The EPA is partnering with the USGS, NOAA, the U.S. Army, the Water Environment Federation, and Greater Cincinnati Water Works to solicit development of an innovative, biologically based effects monitor/biosensor that responds to and measures groups of environmental stressors in water (e.g., hepatic toxicants, endocrine disrupting compounds, pesticides, heavy metals, etc.) that are linked to adverse health outcomes. In this case, the sensor is not used to identify a specific chemical(s), but rather to detect and to quantify perturbations in known toxicity pathways during field deployments [25].

Finally, perhaps the best illustration of the benefits that sensor technologies offer society is embodied in the idea of smart cities, in which cities and municipalities use a multitude of sensors that provide real-time information used to dynamically control and optimize conditions. In such a future cityscape, sensors would be deployed to measure traffic conditions, air quality, water quality, mass transit usage, and to control infrastructure assets like traffic lights, combined water and sewer drainage, mass transit, energy demands, etc. [26]. Cities like Chicago are already embracing the idea of this new frontier through the Array of Things initiative led by the Argonne National Laboratory [27]. Efforts such as these showcase the potential sensor technologies and the IoT have in bringing about transformations in quality of life as well as public administration [28].

Given that many environmental issues extend across the domain of multiple government agencies as well as non-governmental entities, leveraging and coordinating along common interests and missions can be a powerful tool for addressing and solving critical needs.

## 3. The Collaborative Role of the Federal Government

The federal government has played an important role in the continued evolution of sensor technology for a wide variety of applications related to source characterization and exposure science. The federal government has the ability to provide overarching guidance and direction that will lead to more effective and targeted investments by the private sector, as well as more consistent and comparable results that enhance the utility of these technologies. Based on our experience thus far, we delineate five priority areas in which coordinated action by both public and private sectors can yield a tremendous return on investment for all the stakeholders involved.

Educating developers of new technology: Whereas sensor developers have expertise in the elements of technology (e.g., electronics, communications, software, etc.), many lack scientific knowledge in the domain in which they intend to operate (e.g., air quality, exposure science). Furthermore, they may also be unfamiliar with application requirements, sampling methodologies, and the end users’ needs. We have learned through our interactions (e.g., challenge competitions) thus far that the government, in conjunction with other stakeholders, can help educate device makers in these areas, thus providing them with valuable input and guidance to assist them in bringing viable products to the marketplace.

Establishing standards for performance: Due to the rapid proliferation of low-cost sensors yielding data of unknown quality, users are often faced with a confusing array of products to choose from, with very little information or guidance on the quality of the data produced by these devices. Government entities at the state, national, and international levels have responded to this critical gap by taking on the task of evaluating the performance of new devices [18,19,29]. The technology, however, is already outpacing the ability of the public sector to track and evaluate it. One option to help alleviate this problem is through the availability of a third-party technology certification program, whereby device makers can submit their technologies to independent laboratories for testing, review, and certification. This option would enable greater confidence in the data generated and allow users to choose sensor devices that conform best to their application needs. Although focused on analytical methods/kits and not sensors per se, an excellent example is the Performance Tested Methods program of the AOAC Research Institute [30], which provides independent third-party review and certification for proprietary test method performance. The public sector’s role in establishing the testing protocols and developing self-sustaining contractual vehicles and mechanisms can help build the confidence of both consumers and governing bodies in the quality of data produced.

Implementing data and metadata standards: Currently, data are generated by sensors in a wide variety of formats and varying data elements and units. Establishing and promoting adherence to a common set of data reporting and exchange formats, as well as a common set of data and metadata elements, will help support the free and open exchange of data, thus maximizing the utility and comparability of data generated by sensors from a variety of manufacturers [31].

Educating on data interpretation: Even if the data being generated are of good quality, extracting actionable information and usable knowledge from a set of numbers is challenging. Providing guidance to both sensor device makers and users on how high-sample-rate data generated by sensors should be interpreted with respect to exposure and potential effects on humans and ecosystems is critical for this market to flourish. For example, users’ interpretation of how near real-time data collected every minute by sensors relates to hourly, daily, or annual standards or to air quality index values is a crucial issue in establishing the usefulness of sensors.

Supporting a sensor data commons: Another area in need of infrastructural investment is a “sensor data commons”, a freely available web-based resource for generators and users of environmental sensor data to upload, store, analyze, visualize, and share data collected from sensors. Limitations on access and openness at the data layer reduce the utility of data to society as a whole. However, several challenges exist when considering such an infrastructure, including: (1) cost of development and maintenance for an ever-growing data repository, (2) need for QA/QC of the data being made available, (3) complexity of the data and analytics necessary to extract useful information and the need to develop an interoperable ontology, and (4) issues of data ownership, privacy, and security. Some or all of these areas would likely be addressed without the federal government’s involvement and eventually may even converge to the same endpoint, but the federal government can expedite this convergence and minimize inefficiencies, duplications, and investments that are “off target”. In addition, consistent guidance from the federal government can also reduce the need to expend federal, state, and local resources to respond to misapplication and misinterpretation of sensor technology.

## 4. Moving Forward

Rapid technology development is not only disruptive in the private sector for the consumer and commercial space, but also is challenging for the government sector. Environmental sensing is being viewed as a technology that could greatly benefit from a more coordinated effort on the part of multiple governmental entities in order to maximize its full potential. This is certainly the case with environmental sensors, a new breed of technologies for monitoring the quality of the environment and pollution levels in near real-time. Most low-cost sensors available on the market today incorporate both sampling and analysis in one instrument, allowing for near real-time measurements. While some traditional instruments also allow for higher time resolution measurement, the critical difference between them and the new sensor instruments lies in the cost and data quality. Higher time resolution reference-grade instruments are much costlier and involve a much higher level of quality assurance, producing more confidence in the data generated. Currently, low-cost instruments typically lack rigorous data quality checks and thus produce data of lower or unknown quality. Given that data of low or unknown quality, is nearly useless or worse, misleading, part of the government’s role should be to cooperate with makers of low-cost sensors to enable the inclusion of data quality elements that provide users the ability to determine the level of uncertainty associated with sensor data and which applications they might support.

While infrastructural work in a sensor data commons and many of the other areas discussed above can be greatly influenced and incentivized by investment and coordination across the public sector, future developments should comply with the FAIR (Findable, Accessible, Interoperable, Re-usable) data principle [32,33]. To be Findable means that (1) data/metadata are given a globally unique link to eternally persistent identifiers, (2) the metadata provides proper and adequate description of the data allowing users to discern the appropriate use of the data, and (3) the metadata are registered and indexed in a searchable resource. To be Accessible implies that data/metadata are retrievable by their identifiers using commonly used communication protocols. To be Interoperable means that the data/metadata either adhere to or reference established domain vocabularies and terminologies or formally establish their own. Finally, to be Re-usable implies that there is a license to use and reuse the data, and that the metadata are rich enough to describe the provenance of the data and the other information that allows downstream users to perform re-analysis, meta-analysis, and other uses for which the data were not originally collected or intended. From a public perspective, it would be undesirable, counterproductive, and detrimental to the greater public good if each sensor device maker chose to develop solely within its own proprietary platform with tight control and limitations on the data. In such a case, data accessibility and cross-comparison of data among sensors in a heterogeneous deployment would be nearly impossible. The ability to freely share data is what makes these technologies so potentially powerful and compelling.

An excellent example of this type of infrastructural work already underway is from the Woods Hole Oceanographic Institute (WHOI), which was awarded an Earth Cube [34] grant by the National Science Foundation (NSF), Geosciences Directorate and the Directorate for Computer and Information Science and Engineering’s Division of Advanced Cyber infrastructure to help advance data-sharing in an open, transparent, and inclusive manner to accelerate knowledge development. The WHOI research program, called X-DOMES [35,36], for Cross-Domain Observational Metadata for Environmental Sensing, seeks to establish community-based data and metadata standards for environmental sensing. An additional goal is to use and incorporate existing ontologies to fully describe the domain(s) encompassing this effort and make it fully computable in a semantic web framework. Although many scientific disciplines now have formalized controlled vocabularies and ontologies, the later aspect of implementing them within a semantic framework is significant in both the effort involved as well as the benefit derived. The advantage of a semantic web implementation allows improved data-focused services and built-in intelligence. This effort seeks to provide a unifying approach to describing sensors and observations across geo-science domains. With much of the heavy lifting being accomplished under this effort, ES21 hopes to leverage this work as the core architecture for advancing sensing in the context of exposure science.

Another significant trend eagerly anticipated by epidemiologists, exposure scientists, the medical community, and others working in environmental health, is the convergence of technologies that sense environmental conditions (e.g., air sensors) with those that sense and track physiologic responses. Devices like Fitbit and Apple Watch, and their associated health “apps” now comprise a multibillion-dollar business in what is termed mobile health, or mHealth. The maturation of these technologies and the confluence of simultaneous data streams from both sets of devices offers the potential to revolutionize our understanding of public health and treatment of disease. Achieving that goal; however, will require a level of harmonization between these technologies, the development of compatible data architectures and infrastructures able to ingest both distinct data streams, and the analytics and computing intelligence to make sense of it all. The National Institutes of Health (NIH), Big Data to Knowledge [37], and Mobile Sensor Data to Knowledge [38] are efforts aimed at resolving some of these issues by developing some of the software and standards to achieve these goals. While these efforts are primarily led by NIH and the research grants involved, the active participation of other government institutions is essential to their success. Close coordination with agencies and other grantee institutions like the NSF will ensure better, more rapid research outputs and more beneficial community outcomes.

The ability of federal agencies and granting institutions like NIH and NSF to better coordinate their funding and require that the outputs of that research and data be readily available to the public will allow research and progress to advance more rapidly. Just as with the SBIR work, a more coordinated effort in funding research will likely yield better, more useful results and allow institutions to reduce redundancy and be more strategic in the funding they do pursue.

Finally, the federal family of institutions must further emphasize the public nature of the data generated within government and from government-funded research, and incentivize the timely release of such data. Currently, research dollars are predominately used to conduct the research. Few of the resources are used for scientific data management, documentation, and public release and access. Cross-governmental coordination will be essential in revamping the structure of federal research grants to incentivize and prioritize the public nature of data within the granting process and to realize the goals of “open” and “FAIR” data.
